# Dichlorido[2-(3,5-dimethyl-1*H*-pyrazol-1-yl-κ*N* ^2^)-1,10-phenanthroline-κ^2^ *N*,*N*′]cadmium(II)

**DOI:** 10.1107/S1600536810016910

**Published:** 2010-05-15

**Authors:** You Min Sun, Yu Qing Wang, Hui-Xue Ren

**Affiliations:** a School of Municipal and Environmental Engineering, Shandong Jianzhu University, Chemical Engineering and Materials Science, Jinan 250101, People’s Republic of China; b Department of Chemistry, Shandong Normal University, Jinan 250014, People’s Republic of China

## Abstract

The asymmetric unit of the title compound, [CdCl_2_(C_17_H_14_N_4_)], contains two independent mol­ecules in which the Cd^II^ ions are in distorted trigonal-bipyramidal CdN_3_Cl_2_ coordination environments. In the crystal structure, there is a π–π stacking inter­action involving a pyridine ring and a symmetry-related benzene ring, with a centroid–centroid distance of 3.5088 (19) Å.

## Related literature

For a related structure, see: Wang *et al.* (2009 ▶).
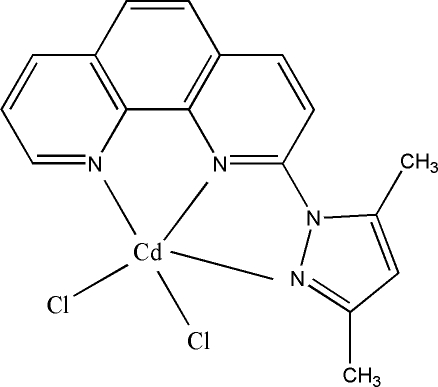


## Experimental

### 

#### Crystal data

[CdCl_2_(C_17_H_14_N_4_)]
*M* *_r_* = 457.63
Triclinic, 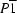

*a* = 10.6268 (12) Å
*b* = 10.7903 (12) Å
*c* = 15.6828 (17) Å
α = 84.220 (2)°
β = 80.051 (2)°
γ = 74.864 (1)°
*V* = 1706.9 (3) Å^3^
*Z* = 4
Mo *K*α radiation
μ = 1.60 mm^−1^
*T* = 298 K
0.36 × 0.25 × 0.19 mm

#### Data collection

Bruker SMART APEX CCD diffractometer
Absorption correction: multi-scan (*SADABS*; Sheldrick, 1996 ▶) *T*_min_ = 0.597, *T*_max_ = 0.751
9365 measured reflections
6562 independent reflections
5658 reflections with *I* > 2σ(*I*)
*R*_int_ = 0.017

#### Refinement

*R*[*F* ^2^ > 2σ(*F* ^2^)] = 0.029
*wR*(*F* ^2^) = 0.078
*S* = 1.05
6562 reflections
437 parameters
H-atom parameters constrained
Δρ_max_ = 0.44 e Å^−3^
Δρ_min_ = −0.57 e Å^−3^

### 

Data collection: *SMART* (Bruker, 1997 ▶); cell refinement: *SAINT* (Bruker, 1997 ▶); data reduction: *SAINT*; program(s) used to solve structure: *SHELXTL* (Sheldrick, 2008 ▶); program(s) used to refine structure: *SHELXTL*; molecular graphics: *SHELXTL*; software used to prepare material for publication: *SHELXTL*.

## Supplementary Material

Crystal structure: contains datablocks I, global. DOI: 10.1107/S1600536810016910/lh5038sup1.cif

Structure factors: contains datablocks I. DOI: 10.1107/S1600536810016910/lh5038Isup2.hkl

Additional supplementary materials:  crystallographic information; 3D view; checkCIF report

## Figures and Tables

**Fig. 1.**
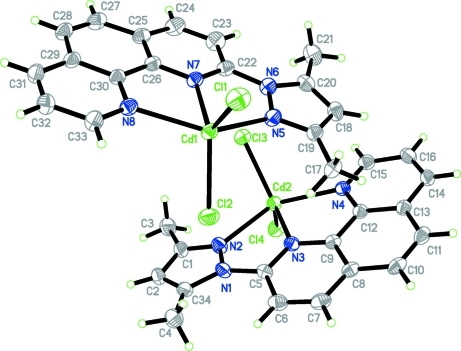
The asymmetric unit of the title compound with the atom-numbering scheme. Displacement ellipsoids are drawn at the 30% probability level.

## supplementary crystallographic information

### Comment

Derivatives of 1,10-phenanthroline play an important role in modern coordination chemistry and many complexes have been reported with these types of compounds as ligands, but to date only one other structure has been reported which contains the ligand 2-(3,5-Dimethyl-1H-pyrazol-1-yl)-1,10-phenanthroline (Wang et al.,  2009). Herein we report the crystal structure of the title complex (I).

The asymmetric unit of the title complex in shown in Fig. 1. There are two independent molecules in the asymmetric unit. The Cd^II^ ions are coordinated by three N atoms and two chloride ligands in distorted trigonal bipyramidal geometries. This coordination geometry is essentially the same as in the previously reported Cd^II^ complex (Wang et al.,  2009). Generally, the Cd^II^ ion assumes a six atom coordination mode but the coordination in the title complex may be attributed to the chelation mode of the 2-(3,5-Dimethyl-1H-pyrazol-1-yl)-1,10-phenanthroline ligand. In the crystal structure, there is a π–π stacking interaction involving the pyridine ring and a symmetry related benzene ring with the relevant distances being Cg1···Cg2^i^ = 3.5088 (19) Å and Cg1···Cg2^i^_perp_ = 3.461 Å (symmetry code: (i) 1-x, 2-y, -z; Cg1 and Cg2 are the centroids of C29-C33/N8 pyridine ring and C25-C30 benzene ring, respectively; Cg1···Cg2^i^_perp_ is the perpendicular distance from Cg1 ring to Cg2^i^ ring).

### Experimental

A 10 ml methanol solution of 2-(3,5-Dimethyl-1*H*-pyrazol-1-yl)-1,10-phenanthroline (0.0539 g, 0.196 mmol) was added into 10 ml H_2_O solution containing CdCl_2_2.56H_2_O (0.0459 g, 0.201 mmol), and the mixed soluton was stirred for a few minutes. The colorless single crystals were obtained after the filtrate had been allowed to stand at room temperature for about a week.

### Refinement

All H atoms were placed in calculated positions and refined as riding with C—H = 0.96 Å, *U*_iso_ = 1.5*U*_eq_(C) for methyl H and C—H = 0.93 Å, *U*_iso_ = 1.2*U*_eq_(C) for other H atoms.

### Crystal data

**Table:**

[CdCl_2_(C_17_H_14_N_4_)]	*Z* = 4
*M**_r_* = 457.63	*F*(000) = 904
Triclinic, *P*1	*D*_x_ = 1.781 Mg m^−^^3^
Hall symbol: -P 1	Mo *K*α radiation, λ = 0.71073 Å
*a* = 10.6268 (12) Å	Cell parameters from 5552 reflections
*b* = 10.7903 (12) Å	θ = 2.4–28.1°
*c* = 15.6828 (17) Å	µ = 1.60 mm^−^^1^
α = 84.220 (2)°	*T* = 298 K
β = 80.051 (2)°	Block, colorless
γ = 74.864 (1)°	0.36 × 0.25 × 0.19 mm
*V* = 1706.9 (3) Å^3^	

### Data collection

**Table:**

Bruker SMART APEX CCD diffractometer	6562 independent reflections
Radiation source: fine-focus sealed tube	5658 reflections with *I* > 2σ(*I*)
graphite	*R*_int_ = 0.017
φ and ω scans	θ_max_ = 26.0°, θ_min_ = 2.0°
Absorption correction: multi-scan (*SADABS*; Sheldrick, 1996)	*h* = −13→12
*T*_min_ = 0.597, *T*_max_ = 0.751	*k* = −13→12
9365 measured reflections	*l* = −14→19

### Refinement

**Table:**

Refinement on *F*^2^	Primary atom site location: structure-invariant direct methods
Least-squares matrix: full	Secondary atom site location: difference Fourier map
*R*[*F*^2^ > 2σ(*F*^2^)] = 0.029	Hydrogen site location: inferred from neighbouring sites
*wR*(*F*^2^) = 0.078	H-atom parameters constrained
*S* = 1.05	*w* = 1/[σ^2^(*F*_o_^2^) + (0.0404*P*)^2^ + 0.2293*P*] where *P* = (*F*_o_^2^ + 2*F*_c_^2^)/3
6562 reflections	(Δ/σ)_max_ = 0.099
437 parameters	Δρ_max_ = 0.44 e Å^−^^3^
0 restraints	Δρ_min_ = −0.57 e Å^−^^3^

### Special details

**Table:**

Geometry. All esds (except the esd in the dihedral angle between two l.s. planes) are estimated using the full covariance matrix. The cell esds are taken into account individually in the estimation of esds in distances, angles and torsion angles; correlations between esds in cell parameters are only used when they are defined by crystal symmetry. An approximate (isotropic) treatment of cell esds is used for estimating esds involving l.s. planes.
Refinement. Refinement of *F*^2^ against ALL reflections. The weighted *R*-factor wR and goodness of fit *S* are based on *F*^2^, conventional *R*-factors *R* are based on *F*, with *F* set to zero for negative *F*^2^. The threshold expression of *F*^2^ > σ(*F*^2^) is used only for calculating *R*-factors(gt) etc. and is not relevant to the choice of reflections for refinement. *R*-factors based on *F*^2^ are statistically about twice as large as those based on *F*, and *R*- factors based on ALL data will be even larger.

### Fractional atomic coordinates and isotropic or equivalent isotropic displacement parameters (Å^2^)

**Table:**

	*x*	*y*	*z*	*U*_iso_*/*U*_eq_	
C1	0.0105 (3)	0.8006 (3)	0.1063 (2)	0.0478 (7)	
C2	−0.0464 (3)	0.9331 (3)	0.1130 (2)	0.0538 (8)	
H2	−0.0722	0.9919	0.0678	0.065*	
C3	0.0501 (4)	0.7214 (4)	0.0295 (2)	0.0601 (9)	
H3A	0.1441	0.7022	0.0134	0.090*	
H3B	0.0087	0.7681	−0.0180	0.090*	
H3C	0.0228	0.6427	0.0433	0.090*	
C4	−0.1026 (4)	1.0890 (3)	0.2347 (3)	0.0689 (11)	
H4A	−0.1148	1.1549	0.1888	0.103*	
H4B	−0.0376	1.1007	0.2667	0.103*	
H4C	−0.1846	1.0942	0.2728	0.103*	
C5	0.0030 (3)	0.8096 (3)	0.3282 (2)	0.0390 (6)	
C6	−0.0555 (3)	0.8921 (3)	0.3966 (2)	0.0487 (8)	
H6	−0.1049	0.9752	0.3856	0.058*	
C7	−0.0377 (3)	0.8472 (3)	0.4782 (2)	0.0475 (8)	
H7	−0.0761	0.9003	0.5238	0.057*	
C8	0.0372 (3)	0.7223 (3)	0.49604 (19)	0.0393 (7)	
C9	0.0894 (3)	0.6466 (3)	0.42457 (18)	0.0359 (6)	
C10	0.0607 (3)	0.6677 (3)	0.58028 (19)	0.0466 (8)	
H10	0.0249	0.7171	0.6280	0.056*	
C11	0.1327 (3)	0.5477 (3)	0.5924 (2)	0.0465 (7)	
H11	0.1474	0.5159	0.6481	0.056*	
C12	0.1647 (3)	0.5155 (3)	0.43745 (18)	0.0358 (6)	
C13	0.1877 (3)	0.4668 (3)	0.52115 (18)	0.0399 (7)	
C14	0.2625 (3)	0.3394 (3)	0.5303 (2)	0.0466 (7)	
H14	0.2807	0.3037	0.5846	0.056*	
C15	0.2792 (3)	0.3240 (3)	0.3788 (2)	0.0452 (7)	
H15	0.3106	0.2744	0.3308	0.054*	
C16	0.3081 (3)	0.2685 (3)	0.4597 (2)	0.0500 (8)	
H16	0.3580	0.1841	0.4651	0.060*	
C17	0.3677 (3)	0.7559 (3)	0.4634 (2)	0.0537 (8)	
H17A	0.4227	0.7934	0.4900	0.081*	
H17B	0.3278	0.7012	0.5057	0.081*	
H17C	0.3000	0.8228	0.4415	0.081*	
C18	0.4929 (3)	0.5453 (3)	0.3842 (2)	0.0495 (8)	
H18	0.4745	0.4831	0.4266	0.059*	
C19	0.4497 (3)	0.6781 (3)	0.3903 (2)	0.0428 (7)	
C20	0.5672 (3)	0.5232 (3)	0.3044 (2)	0.0446 (7)	
C21	0.6377 (4)	0.3967 (3)	0.2680 (3)	0.0615 (10)	
H21A	0.6042	0.3880	0.2164	0.092*	
H21B	0.6240	0.3286	0.3100	0.092*	
H21C	0.7304	0.3921	0.2541	0.092*	
C22	0.6184 (3)	0.6828 (3)	0.1799 (2)	0.0416 (7)	
C23	0.6857 (4)	0.5978 (3)	0.1149 (2)	0.0562 (9)	
H23	0.6998	0.5093	0.1258	0.067*	
C24	0.7292 (3)	0.6480 (3)	0.0362 (2)	0.0563 (9)	
H24	0.7725	0.5928	−0.0075	0.068*	
C25	0.7108 (3)	0.7799 (3)	0.0189 (2)	0.0442 (7)	
C26	0.6429 (3)	0.8578 (3)	0.08726 (18)	0.0378 (6)	
C27	0.7545 (3)	0.8400 (4)	−0.0627 (2)	0.0538 (9)	
H27	0.7975	0.7893	−0.1088	0.065*	
C28	0.7342 (3)	0.9674 (4)	−0.0735 (2)	0.0508 (8)	
H28	0.7657	1.0035	−0.1267	0.061*	
C29	0.6659 (3)	1.0500 (3)	−0.00618 (19)	0.0431 (7)	
C30	0.6183 (3)	0.9951 (3)	0.07433 (18)	0.0372 (6)	
C31	0.6410 (3)	1.1837 (3)	−0.0158 (2)	0.0512 (8)	
H31	0.6708	1.2233	−0.0681	0.061*	
C32	0.5733 (3)	1.2558 (3)	0.0512 (2)	0.0543 (8)	
H32	0.5575	1.3449	0.0458	0.065*	
C33	0.5276 (3)	1.1942 (3)	0.1286 (2)	0.0493 (8)	
H33	0.4791	1.2445	0.1736	0.059*	
C34	−0.0569 (3)	0.9600 (3)	0.1969 (2)	0.0481 (8)	
Cd1	0.48003 (2)	0.95308 (2)	0.270728 (13)	0.03849 (8)	
Cd2	0.14567 (2)	0.54306 (2)	0.234337 (13)	0.03963 (8)	
Cl1	0.60242 (9)	1.01735 (10)	0.36848 (6)	0.0642 (2)	
Cl2	0.24121 (8)	1.03443 (9)	0.30304 (5)	0.0550 (2)	
Cl3	0.35931 (8)	0.52714 (9)	0.14531 (6)	0.0584 (2)	
Cl4	0.01029 (9)	0.42021 (9)	0.18715 (5)	0.0559 (2)	
N1	−0.0097 (2)	0.8449 (2)	0.24047 (17)	0.0433 (6)	
N2	0.0327 (3)	0.7477 (2)	0.18384 (17)	0.0463 (6)	
N3	0.0710 (2)	0.6911 (2)	0.34320 (15)	0.0369 (5)	
N4	0.2094 (2)	0.4436 (2)	0.36694 (15)	0.0376 (5)	
N5	0.4937 (2)	0.7375 (2)	0.31787 (16)	0.0433 (6)	
N6	0.5672 (2)	0.6416 (2)	0.26423 (16)	0.0419 (6)	
N7	0.5973 (2)	0.8080 (2)	0.16565 (15)	0.0370 (5)	
N8	0.5498 (2)	1.0681 (2)	0.14081 (16)	0.0409 (6)	

### Atomic displacement parameters (Å^2^)

**Table:**

	*U*^11^	*U*^22^	*U*^33^	*U*^12^	*U*^13^	*U*^23^
C1	0.0436 (17)	0.0536 (19)	0.0458 (18)	−0.0113 (14)	−0.0139 (14)	0.0090 (15)
C2	0.0459 (18)	0.056 (2)	0.055 (2)	−0.0098 (15)	−0.0118 (15)	0.0182 (16)
C3	0.062 (2)	0.068 (2)	0.049 (2)	−0.0108 (18)	−0.0173 (17)	0.0014 (17)
C4	0.076 (3)	0.0362 (18)	0.079 (3)	−0.0009 (17)	0.008 (2)	0.0052 (18)
C5	0.0349 (14)	0.0368 (15)	0.0448 (17)	−0.0063 (12)	−0.0079 (13)	−0.0035 (13)
C6	0.0477 (18)	0.0375 (17)	0.058 (2)	−0.0049 (14)	−0.0033 (15)	−0.0133 (15)
C7	0.0443 (17)	0.0455 (18)	0.053 (2)	−0.0130 (14)	0.0031 (15)	−0.0191 (15)
C8	0.0359 (15)	0.0468 (17)	0.0385 (16)	−0.0162 (13)	−0.0001 (12)	−0.0112 (13)
C9	0.0327 (14)	0.0409 (16)	0.0369 (15)	−0.0125 (12)	−0.0043 (12)	−0.0087 (12)
C10	0.0434 (17)	0.064 (2)	0.0369 (16)	−0.0223 (16)	0.0043 (13)	−0.0168 (15)
C11	0.0452 (17)	0.067 (2)	0.0331 (16)	−0.0233 (16)	−0.0056 (13)	−0.0040 (15)
C12	0.0320 (14)	0.0416 (16)	0.0366 (15)	−0.0134 (12)	−0.0062 (12)	−0.0016 (12)
C13	0.0341 (15)	0.0522 (18)	0.0371 (16)	−0.0176 (13)	−0.0062 (12)	−0.0004 (13)
C14	0.0400 (16)	0.059 (2)	0.0425 (18)	−0.0159 (14)	−0.0125 (14)	0.0110 (15)
C15	0.0429 (17)	0.0401 (17)	0.0505 (19)	−0.0078 (13)	−0.0032 (14)	−0.0062 (14)
C16	0.0419 (17)	0.0450 (18)	0.062 (2)	−0.0092 (14)	−0.0122 (16)	0.0068 (16)
C17	0.0515 (19)	0.061 (2)	0.0455 (19)	−0.0177 (16)	0.0027 (15)	0.0041 (16)
C18	0.0538 (19)	0.0452 (18)	0.056 (2)	−0.0240 (15)	−0.0173 (16)	0.0109 (15)
C19	0.0390 (16)	0.0489 (18)	0.0430 (17)	−0.0175 (13)	−0.0064 (13)	0.0037 (14)
C20	0.0441 (17)	0.0384 (16)	0.056 (2)	−0.0122 (13)	−0.0169 (15)	−0.0023 (14)
C21	0.079 (3)	0.0358 (17)	0.072 (2)	−0.0122 (17)	−0.020 (2)	−0.0031 (17)
C22	0.0387 (16)	0.0397 (17)	0.0466 (18)	−0.0106 (13)	−0.0026 (13)	−0.0076 (14)
C23	0.064 (2)	0.0377 (17)	0.064 (2)	−0.0087 (15)	0.0013 (18)	−0.0145 (16)
C24	0.060 (2)	0.055 (2)	0.051 (2)	−0.0104 (17)	0.0038 (17)	−0.0224 (17)
C25	0.0387 (16)	0.0526 (19)	0.0415 (17)	−0.0109 (14)	−0.0017 (13)	−0.0128 (14)
C26	0.0333 (14)	0.0460 (17)	0.0343 (15)	−0.0102 (12)	−0.0038 (12)	−0.0054 (13)
C27	0.0504 (19)	0.074 (3)	0.0352 (17)	−0.0124 (17)	0.0019 (14)	−0.0157 (16)
C28	0.0451 (18)	0.074 (2)	0.0319 (16)	−0.0166 (16)	−0.0009 (13)	−0.0011 (15)
C29	0.0357 (15)	0.059 (2)	0.0357 (16)	−0.0151 (14)	−0.0066 (12)	0.0013 (14)
C30	0.0325 (14)	0.0450 (16)	0.0358 (15)	−0.0119 (12)	−0.0056 (12)	−0.0029 (13)
C31	0.0485 (18)	0.060 (2)	0.0466 (19)	−0.0204 (16)	−0.0090 (15)	0.0117 (16)
C32	0.057 (2)	0.0468 (19)	0.058 (2)	−0.0166 (16)	−0.0083 (17)	0.0063 (16)
C33	0.057 (2)	0.0434 (18)	0.0464 (18)	−0.0148 (15)	−0.0002 (15)	−0.0054 (14)
C34	0.0359 (16)	0.0398 (17)	0.063 (2)	−0.0065 (13)	−0.0025 (15)	0.0074 (15)
Cd1	0.04044 (13)	0.03712 (13)	0.03636 (13)	−0.01020 (9)	0.00171 (9)	−0.00644 (9)
Cd2	0.04436 (13)	0.03821 (13)	0.03451 (13)	−0.00638 (9)	−0.00465 (9)	−0.00639 (9)
Cl1	0.0600 (5)	0.0840 (7)	0.0571 (5)	−0.0265 (5)	−0.0122 (4)	−0.0152 (5)
Cl2	0.0421 (4)	0.0694 (6)	0.0472 (5)	−0.0050 (4)	0.0000 (3)	−0.0101 (4)
Cl3	0.0481 (4)	0.0586 (5)	0.0576 (5)	−0.0058 (4)	0.0042 (4)	0.0072 (4)
Cl4	0.0632 (5)	0.0629 (5)	0.0477 (5)	−0.0248 (4)	−0.0041 (4)	−0.0153 (4)
N1	0.0424 (14)	0.0364 (13)	0.0478 (15)	−0.0027 (11)	−0.0101 (12)	0.0000 (11)
N2	0.0522 (15)	0.0404 (14)	0.0442 (15)	−0.0040 (12)	−0.0117 (12)	−0.0044 (12)
N3	0.0367 (12)	0.0344 (13)	0.0393 (13)	−0.0067 (10)	−0.0065 (10)	−0.0048 (10)
N4	0.0377 (12)	0.0359 (13)	0.0384 (13)	−0.0078 (10)	−0.0036 (10)	−0.0060 (10)
N5	0.0473 (14)	0.0365 (13)	0.0438 (15)	−0.0109 (11)	0.0015 (12)	−0.0039 (11)
N6	0.0465 (14)	0.0344 (13)	0.0459 (15)	−0.0130 (11)	−0.0040 (12)	−0.0045 (11)
N7	0.0377 (12)	0.0355 (13)	0.0372 (13)	−0.0099 (10)	0.0001 (10)	−0.0071 (10)
N8	0.0408 (13)	0.0401 (14)	0.0417 (14)	−0.0112 (11)	−0.0012 (11)	−0.0068 (11)

### Geometric parameters (Å, °)

**Table:**

C1—N2	1.322 (4)	C19—N5	1.321 (4)
C1—C2	1.407 (5)	C20—N6	1.366 (4)
C1—C3	1.485 (5)	C20—C21	1.492 (4)
C2—C34	1.356 (5)	C21—H21A	0.9600
C2—H2	0.9300	C21—H21B	0.9600
C3—H3A	0.9600	C21—H21C	0.9600
C3—H3B	0.9600	C22—N7	1.313 (4)
C3—H3C	0.9600	C22—C23	1.410 (4)
C4—C34	1.496 (5)	C22—N6	1.414 (4)
C4—H4A	0.9600	C23—C24	1.353 (5)
C4—H4B	0.9600	C23—H23	0.9300
C4—H4C	0.9600	C24—C25	1.390 (5)
C5—N3	1.314 (4)	C24—H24	0.9300
C5—C6	1.415 (4)	C25—C26	1.406 (4)
C5—N1	1.410 (4)	C25—C27	1.435 (5)
C6—C7	1.349 (5)	C26—N7	1.354 (4)
C6—H6	0.9300	C26—C30	1.435 (4)
C7—C8	1.403 (4)	C27—C28	1.333 (5)
C7—H7	0.9300	C27—H27	0.9300
C8—C9	1.401 (4)	C28—C29	1.422 (4)
C8—C10	1.427 (4)	C28—H28	0.9300
C9—N3	1.346 (4)	C29—C31	1.395 (5)
C9—C12	1.445 (4)	C29—C30	1.409 (4)
C10—C11	1.335 (5)	C30—N8	1.357 (3)
C10—H10	0.9300	C31—C32	1.357 (5)
C11—C13	1.434 (4)	C31—H31	0.9300
C11—H11	0.9300	C32—C33	1.397 (4)
C12—N4	1.360 (3)	C32—H32	0.9300
C12—C13	1.401 (4)	C33—N8	1.318 (4)
C13—C14	1.404 (4)	C33—H33	0.9300
C14—C16	1.355 (5)	C34—N1	1.372 (4)
C14—H14	0.9300	Cd1—N5	2.344 (2)
C15—N4	1.322 (4)	Cd1—N7	2.347 (2)
C15—C16	1.396 (5)	Cd1—N8	2.386 (3)
C15—H15	0.9300	Cd1—Cl1	2.4283 (9)
C16—H16	0.9300	Cd1—Cl2	2.4393 (8)
C17—C19	1.499 (4)	Cd2—N3	2.348 (2)
C17—H17A	0.9600	Cd2—N2	2.353 (3)
C17—H17B	0.9600	Cd2—N4	2.365 (2)
C17—H17C	0.9600	Cd2—Cl3	2.4254 (9)
C18—C20	1.366 (5)	Cd2—Cl4	2.4365 (8)
C18—C19	1.394 (4)	N1—N2	1.376 (3)
C18—H18	0.9300	N5—N6	1.384 (3)
			
N2—C1—C2	109.0 (3)	C24—C23—H23	120.8
N2—C1—C3	120.3 (3)	C22—C23—H23	120.8
C2—C1—C3	130.6 (3)	C23—C24—C25	121.7 (3)
C34—C2—C1	107.9 (3)	C23—C24—H24	119.2
C34—C2—H2	126.1	C25—C24—H24	119.2
C1—C2—H2	126.1	C24—C25—C26	116.3 (3)
C1—C3—H3A	109.5	C24—C25—C27	124.8 (3)
C1—C3—H3B	109.5	C26—C25—C27	118.9 (3)
H3A—C3—H3B	109.5	N7—C26—C25	122.2 (3)
C1—C3—H3C	109.5	N7—C26—C30	118.1 (2)
H3A—C3—H3C	109.5	C25—C26—C30	119.7 (3)
H3B—C3—H3C	109.5	C28—C27—C25	121.0 (3)
C34—C4—H4A	109.5	C28—C27—H27	119.5
C34—C4—H4B	109.5	C25—C27—H27	119.5
H4A—C4—H4B	109.5	C27—C28—C29	122.0 (3)
C34—C4—H4C	109.5	C27—C28—H28	119.0
H4A—C4—H4C	109.5	C29—C28—H28	119.0
H4B—C4—H4C	109.5	C31—C29—C30	117.7 (3)
N3—C5—C6	121.4 (3)	C31—C29—C28	123.4 (3)
N3—C5—N1	114.9 (2)	C30—C29—C28	118.9 (3)
C6—C5—N1	123.7 (3)	N8—C30—C29	122.1 (3)
C7—C6—C5	118.4 (3)	N8—C30—C26	118.5 (2)
C7—C6—H6	120.8	C29—C30—C26	119.5 (3)
C5—C6—H6	120.8	C32—C31—C29	119.8 (3)
C6—C7—C8	121.6 (3)	C32—C31—H31	120.1
C6—C7—H7	119.2	C29—C31—H31	120.1
C8—C7—H7	119.2	C31—C32—C33	119.0 (3)
C9—C8—C7	116.1 (3)	C31—C32—H32	120.5
C9—C8—C10	118.8 (3)	C33—C32—H32	120.5
C7—C8—C10	125.0 (3)	N8—C33—C32	123.2 (3)
N3—C9—C8	122.3 (3)	N8—C33—H33	118.4
N3—C9—C12	118.0 (2)	C32—C33—H33	118.4
C8—C9—C12	119.7 (3)	C2—C34—N1	106.0 (3)
C11—C10—C8	121.7 (3)	C2—C34—C4	127.7 (3)
C11—C10—H10	119.1	N1—C34—C4	126.2 (3)
C8—C10—H10	119.1	N5—Cd1—N7	66.71 (8)
C10—C11—C13	121.3 (3)	N5—Cd1—N8	136.81 (8)
C10—C11—H11	119.4	N7—Cd1—N8	70.12 (8)
C13—C11—H11	119.4	N5—Cd1—Cl1	101.73 (7)
N4—C12—C13	122.5 (3)	N7—Cd1—Cl1	118.21 (6)
N4—C12—C9	118.0 (2)	N8—Cd1—Cl1	99.48 (6)
C13—C12—C9	119.5 (3)	N5—Cd1—Cl2	98.31 (7)
C12—C13—C14	117.4 (3)	N7—Cd1—Cl2	126.95 (6)
C12—C13—C11	118.9 (3)	N8—Cd1—Cl2	106.32 (6)
C14—C13—C11	123.7 (3)	Cl1—Cd1—Cl2	114.58 (3)
C16—C14—C13	119.9 (3)	N3—Cd2—N2	66.81 (8)
C16—C14—H14	120.1	N3—Cd2—N4	70.35 (8)
C13—C14—H14	120.1	N2—Cd2—N4	137.15 (8)
N4—C15—C16	123.3 (3)	N3—Cd2—Cl3	120.27 (6)
N4—C15—H15	118.4	N2—Cd2—Cl3	99.63 (7)
C16—C15—H15	118.4	N4—Cd2—Cl3	100.85 (6)
C14—C16—C15	119.0 (3)	N3—Cd2—Cl4	124.42 (6)
C14—C16—H16	120.5	N2—Cd2—Cl4	97.38 (7)
C15—C16—H16	120.5	N4—Cd2—Cl4	107.40 (6)
C19—C17—H17A	109.5	Cl3—Cd2—Cl4	114.71 (3)
C19—C17—H17B	109.5	C34—N1—N2	110.2 (3)
H17A—C17—H17B	109.5	C34—N1—C5	133.2 (3)
C19—C17—H17C	109.5	N2—N1—C5	116.6 (2)
H17A—C17—H17C	109.5	C1—N2—N1	106.8 (3)
H17B—C17—H17C	109.5	C1—N2—Cd2	133.9 (2)
C20—C18—C19	107.4 (3)	N1—N2—Cd2	118.69 (18)
C20—C18—H18	126.3	C5—N3—C9	120.2 (2)
C19—C18—H18	126.3	C5—N3—Cd2	122.30 (19)
N5—C19—C18	110.1 (3)	C9—N3—Cd2	117.18 (18)
N5—C19—C17	119.5 (3)	C15—N4—C12	117.9 (3)
C18—C19—C17	130.4 (3)	C15—N4—Cd2	125.92 (19)
C18—C20—N6	106.0 (3)	C12—N4—Cd2	116.11 (18)
C18—C20—C21	127.8 (3)	C19—N5—N6	106.0 (2)
N6—C20—C21	126.2 (3)	C19—N5—Cd1	134.7 (2)
C20—C21—H21A	109.5	N6—N5—Cd1	119.24 (17)
C20—C21—H21B	109.5	C20—N6—N5	110.4 (2)
H21A—C21—H21B	109.5	C20—N6—C22	133.3 (3)
C20—C21—H21C	109.5	N5—N6—C22	116.2 (2)
H21A—C21—H21C	109.5	C22—N7—C26	119.7 (2)
H21B—C21—H21C	109.5	C22—N7—Cd1	122.74 (19)
N7—C22—C23	121.7 (3)	C26—N7—Cd1	117.51 (18)
N7—C22—N6	114.9 (2)	C33—N8—C30	118.2 (3)
C23—C22—N6	123.5 (3)	C33—N8—Cd1	126.0 (2)
C24—C23—C22	118.4 (3)	C30—N8—Cd1	115.84 (18)
			
N2—C1—C2—C34	0.7 (4)	C6—C5—N3—C9	−1.8 (4)
C3—C1—C2—C34	−176.7 (3)	N1—C5—N3—C9	−179.9 (2)
N3—C5—C6—C7	1.4 (5)	C6—C5—N3—Cd2	171.8 (2)
N1—C5—C6—C7	179.2 (3)	N1—C5—N3—Cd2	−6.3 (3)
C5—C6—C7—C8	0.4 (5)	C8—C9—N3—C5	0.5 (4)
C6—C7—C8—C9	−1.5 (4)	C12—C9—N3—C5	179.9 (2)
C6—C7—C8—C10	179.9 (3)	C8—C9—N3—Cd2	−173.3 (2)
C7—C8—C9—N3	1.1 (4)	C12—C9—N3—Cd2	6.1 (3)
C10—C8—C9—N3	179.7 (3)	N2—Cd2—N3—C5	1.7 (2)
C7—C8—C9—C12	−178.3 (3)	N4—Cd2—N3—C5	−179.3 (2)
C10—C8—C9—C12	0.3 (4)	Cl3—Cd2—N3—C5	89.4 (2)
C9—C8—C10—C11	1.2 (4)	Cl4—Cd2—N3—C5	−81.2 (2)
C7—C8—C10—C11	179.7 (3)	N2—Cd2—N3—C9	175.4 (2)
C8—C10—C11—C13	−1.3 (5)	N4—Cd2—N3—C9	−5.52 (18)
N3—C9—C12—N4	−1.9 (4)	Cl3—Cd2—N3—C9	−96.85 (19)
C8—C9—C12—N4	177.5 (2)	Cl4—Cd2—N3—C9	92.54 (19)
N3—C9—C12—C13	178.9 (2)	C16—C15—N4—C12	0.4 (4)
C8—C9—C12—C13	−1.7 (4)	C16—C15—N4—Cd2	−176.6 (2)
N4—C12—C13—C14	1.4 (4)	C13—C12—N4—C15	−1.3 (4)
C9—C12—C13—C14	−179.4 (2)	C9—C12—N4—C15	179.5 (3)
N4—C12—C13—C11	−177.6 (2)	C13—C12—N4—Cd2	176.0 (2)
C9—C12—C13—C11	1.6 (4)	C9—C12—N4—Cd2	−3.2 (3)
C10—C11—C13—C12	−0.1 (4)	N3—Cd2—N4—C15	−178.5 (3)
C10—C11—C13—C14	−179.1 (3)	N2—Cd2—N4—C15	−177.2 (2)
C12—C13—C14—C16	−0.6 (4)	Cl3—Cd2—N4—C15	−60.0 (2)
C11—C13—C14—C16	178.3 (3)	Cl4—Cd2—N4—C15	60.4 (2)
C13—C14—C16—C15	−0.2 (4)	N3—Cd2—N4—C12	4.46 (18)
N4—C15—C16—C14	0.3 (5)	N2—Cd2—N4—C12	5.7 (2)
C20—C18—C19—N5	0.3 (4)	Cl3—Cd2—N4—C12	122.91 (18)
C20—C18—C19—C17	−178.7 (3)	Cl4—Cd2—N4—C12	−116.68 (18)
C19—C18—C20—N6	−0.1 (3)	C18—C19—N5—N6	−0.3 (3)
C19—C18—C20—C21	178.4 (3)	C17—C19—N5—N6	178.8 (3)
N7—C22—C23—C24	0.1 (5)	C18—C19—N5—Cd1	−177.2 (2)
N6—C22—C23—C24	179.3 (3)	C17—C19—N5—Cd1	1.9 (5)
C22—C23—C24—C25	1.0 (5)	N7—Cd1—N5—C19	−179.4 (3)
C23—C24—C25—C26	−0.9 (5)	N8—Cd1—N5—C19	−177.5 (3)
C23—C24—C25—C27	179.8 (3)	Cl1—Cd1—N5—C19	64.7 (3)
C24—C25—C26—N7	−0.2 (4)	Cl2—Cd1—N5—C19	−52.7 (3)
C27—C25—C26—N7	179.1 (3)	N7—Cd1—N5—N6	4.00 (19)
C24—C25—C26—C30	−179.2 (3)	N8—Cd1—N5—N6	6.0 (3)
C27—C25—C26—C30	0.1 (4)	Cl1—Cd1—N5—N6	−111.87 (19)
C24—C25—C27—C28	−179.1 (3)	Cl2—Cd1—N5—N6	130.76 (19)
C26—C25—C27—C28	1.6 (5)	C18—C20—N6—N5	−0.1 (3)
C25—C27—C28—C29	−1.7 (5)	C21—C20—N6—N5	−178.6 (3)
C27—C28—C29—C31	−178.8 (3)	C18—C20—N6—C22	−175.3 (3)
C27—C28—C29—C30	0.0 (5)	C21—C20—N6—C22	6.2 (5)
C31—C29—C30—N8	0.4 (4)	C19—N5—N6—C20	0.2 (3)
C28—C29—C30—N8	−178.5 (3)	Cd1—N5—N6—C20	177.69 (18)
C31—C29—C30—C26	−179.4 (3)	C19—N5—N6—C22	176.3 (3)
C28—C29—C30—C26	1.7 (4)	Cd1—N5—N6—C22	−6.2 (3)
N7—C26—C30—N8	−0.6 (4)	N7—C22—N6—C20	179.7 (3)
C25—C26—C30—N8	178.4 (3)	C23—C22—N6—C20	0.5 (5)
N7—C26—C30—C29	179.3 (3)	N7—C22—N6—N5	4.7 (4)
C25—C26—C30—C29	−1.7 (4)	C23—C22—N6—N5	−174.5 (3)
C30—C29—C31—C32	0.1 (4)	C23—C22—N7—C26	−1.3 (4)
C28—C29—C31—C32	179.0 (3)	N6—C22—N7—C26	179.6 (2)
C29—C31—C32—C33	−1.1 (5)	C23—C22—N7—Cd1	178.0 (2)
C31—C32—C33—N8	1.8 (5)	N6—C22—N7—Cd1	−1.1 (4)
C1—C2—C34—N1	−1.3 (4)	C25—C26—N7—C22	1.3 (4)
C1—C2—C34—C4	175.3 (3)	C30—C26—N7—C22	−179.7 (3)
C2—C34—N1—N2	1.4 (3)	C25—C26—N7—Cd1	−178.0 (2)
C4—C34—N1—N2	−175.3 (3)	C30—C26—N7—Cd1	0.9 (3)
C2—C34—N1—C5	−178.7 (3)	N5—Cd1—N7—C22	−1.5 (2)
C4—C34—N1—C5	4.6 (5)	N8—Cd1—N7—C22	180.0 (2)
N3—C5—N1—C34	−170.7 (3)	Cl1—Cd1—N7—C22	89.7 (2)
C6—C5—N1—C34	11.3 (5)	Cl2—Cd1—N7—C22	−84.2 (2)
N3—C5—N1—N2	9.2 (4)	N5—Cd1—N7—C26	177.9 (2)
C6—C5—N1—N2	−168.8 (3)	N8—Cd1—N7—C26	−0.70 (19)
C2—C1—N2—N1	0.1 (3)	Cl1—Cd1—N7—C26	−91.0 (2)
C3—C1—N2—N1	177.8 (3)	Cl2—Cd1—N7—C26	95.15 (19)
C2—C1—N2—Cd2	−171.1 (2)	C32—C33—N8—C30	−1.3 (5)
C3—C1—N2—Cd2	6.7 (5)	C32—C33—N8—Cd1	178.8 (2)
C34—N1—N2—C1	−0.9 (3)	C29—C30—N8—C33	0.2 (4)
C5—N1—N2—C1	179.2 (3)	C26—C30—N8—C33	180.0 (3)
C34—N1—N2—Cd2	171.84 (19)	C29—C30—N8—Cd1	−179.9 (2)
C5—N1—N2—Cd2	−8.1 (3)	C26—C30—N8—Cd1	−0.1 (3)
N3—Cd2—N2—C1	173.9 (3)	N5—Cd1—N8—C33	178.4 (2)
N4—Cd2—N2—C1	172.6 (3)	N7—Cd1—N8—C33	−179.7 (3)
Cl3—Cd2—N2—C1	55.0 (3)	Cl1—Cd1—N8—C33	−63.0 (3)
Cl4—Cd2—N2—C1	−61.8 (3)	Cl2—Cd1—N8—C33	56.2 (3)
N3—Cd2—N2—N1	3.51 (19)	N5—Cd1—N8—C30	−1.5 (3)
N4—Cd2—N2—N1	2.2 (3)	N7—Cd1—N8—C30	0.39 (18)
Cl3—Cd2—N2—N1	−115.4 (2)	Cl1—Cd1—N8—C30	117.09 (19)
Cl4—Cd2—N2—N1	127.9 (2)	Cl2—Cd1—N8—C30	−123.68 (18)
